# Impact of the COVID-19 pandemic on compulsory notification of meningitis during the first wave of the pandemic in Brazil: an ecological study using P-score

**DOI:** 10.1590/1516-3180.2021.0732.15092021

**Published:** 2022-02-23

**Authors:** Carlos Alberto de Oliveira Rocha, Gibson Barros de Almeida Santana, Thiago Cavalcanti Leal, João Paulo Silva de Paiva, Leonardo Feitosa da Silva, Lucas Gomes Santos, Bruno Eduardo Basto Rolim Nunes, Rodrigo Feliciano do Carmo, Carlos Dornels Freire de Souza

**Affiliations:** I Undergraduate Student, Department of Medicine, Center for Studies on Social and Preventive Medicine, Universidade Federal de Alagoas, Arapiraca (AL), Brazil.; II Undergraduate Student, Department of Medicine, Center for Studies on Social and Preventive Medicine, Universidade Federal de Alagoas, Arapiraca (AL), Brazil.; III Undergraduate Student, Department of Medicine, Center for Studies on Social and Preventive Medicine, Universidade Federal de Alagoas, Arapiraca (AL), Brazil.; IV Undergraduate Student, Department of Medicine, Center for Studies on Social and Preventive Medicine, Universidade Federal de Alagoas, Arapiraca (AL), Brazil.; V Undergraduate Student, Department of Medicine, Center for Studies on Social and Preventive Medicine, Universidade Federal de Alagoas, Arapiraca (AL), Brazil.; VI Undergraduate Student, Department of Medicine, Center for Studies on Social and Preventive Medicine, Universidade Federal de Alagoas, Arapiraca (AL), Brazil.; VII Undergraduate Student, Department of Medicine, Center for Studies on Social and Preventive Medicine, Universidade Federal de Alagoas, Arapiraca (AL), Brazil.; VIII PhD. Adjunct Professor, College of Pharmaceutical Sciences; and Adjunct Professor, Postgraduate Program on Biosciences, Health and Biological Sciences, Universidade Federal do Vale do São Francisco (UNIVASF), Petrolina (PE), Brazil.; IX PhD. Adjunct Professor, Department of Medicine; and Professor, Postgraduate Program on Family Health, Universidade Federal de Alagoas (UFAL), Arapiraca (AL), Brazil.

**Keywords:** Epidemiology, Meningitis, Pandemics, Public health, Coronavirus, Disease notification, Neurosciences, Community health, Coronaviruses, COVID-19 virus disease

## Abstract

**BACKGROUND:**

Meningitis is listed as one of the diseases requiring compulsory notification in Brazil. It can affect all age groups and also has no seasonality. Cases can be recorded in all months of the year and in all states of Brazil. Despite its importance, the obligation of immediate notification may have been compromised by the coronavirus disease 2019 (COVID-19) pandemic.

**OBJECTIVE:**

To analyze the immediate impact of the COVID-19 pandemic on compulsory notifications of meningitis in Brazil and its states during the first wave of the pandemic.

**DESIGN AND SETTING:**

This was an ecological study involving all confirmed cases of meningitis in Brazil, in its regions and in its states.

**METHODS:**

Data for the months from 2015 to 2020 were obtained from the database of the Notifiable Diseases Information System (Sistema de Informação de Agravos de Notificação, SINAN), in the Department of Informatics of the National Health System (Departamento de Informática do Sistema Único de Saúde, DATASUS). The P-score was used to obtain the percentage change in the numbers of cases reported in 2020.

**RESULTS:**

A 45.7% reduction in notifications of meningitis in Brazil was observed. Regarding the regions and the states, with the exception of Roraima, all of them showed a negative P-score, with decreasing curves each month.

**CONCLUSION:**

The pandemic caused a negative impact on meningitis notifications in Brazil.

## INTRODUCTION

Coronavirus disease 2019 (COVID-19) was recorded for the first time in Brazil on February 26, 2020. Just over a year later, on June 11, 2021, this country ranked third in the absolute number of disease records (17.2 million cases) and second in the number of deaths (482,000), with incidence of 8190.0/100,000 inhabitants, mortality of 229.4/100,000 and case fatality rate of 2.8%.^1^

Due to the pandemic, the Brazilian healthcare system directed its efforts to combating COVID-19, through adjusting the healthcare network (opening of temporary hospitals and expansion of numbers of infirmary beds and intensive care units, for example), as well as through implementation of non-pharmacological measures to reduce the intensity of disease transmission, such as restricting urban mobility and changes to healthcare services, i.e. reduced hours and numbers of daily appointments.^2^ These measures, although necessary, impacted the diagnosis and management of other endemic diseases in this country, as already observed in investigations involving leprosy^3^ and tuberculosis.^4^ This went against the measures that would be necessary for control and monitoring of these diseases,^5^ which thus received less attention. Consequently, there were delays in diagnosing these diseases, due to the pandemic.^6^

In accordance with Consolidation Ordinance No. 4/GM/MS, of September 28, 2017, meningitis is listed as one of the diseases requiring compulsory notification, given that it is a public health problem.^7^ It is considered endemic in Brazil, can affect all age groups and also has no seasonality. Cases can be recorded in all months of the year and in all states of Brazil.^8^ Despite its importance, the obligation of immediate notification may have been compromised by the COVID-19 pandemic.

In order to analyze the impact of the pandemic on different health problems, tools to quantify this impact have been developed, among which the P-score^9^ stands out. Although this was developed to assess excess mortality during the pandemic, it can also identify underrecording of health events. Moreover, this was the first national study to use P-scores to analyze the impact of the pandemic.

## OBJECTIVE

In the light of the above context, the aim of this study was to analyze the immediate impact of the COVID-19 pandemic on compulsory notifications of meningitis in Brazil and its states during the first wave of the pandemic.

## METHODS

This was an ecological study involving all confirmed cases of meningitis in Brazil, in its regions and states, from 2015 to 2020 The period 2015 to 2019 was used to obtain the expected values of the events analyzed (pre-pandemic period) and the year 2020 was used for comparison purposes (pandemic period). Data were collected in relation to the general population and in relation to children under 15 years of age. The records were obtained from the Notifiable Diseases Information System (Sistema de Informação de Agravos de Notificação, SINAN), of the Department of Informatics of the National Health System (Departamento de Informática do Sistema Único de Saúde, DATASUS).

For analysis, an adaptation of the P-score was applied,^9^ considering the following equation:

$$P\;-\;score=\frac{No.\;of\;meningitis\;cases\;in\;2020\;(January\;-\;August)\;-\;Expected\;meningitis\;case\;numbers\;in\;2020}{Expected\;meningitis\;case\;numbers\;in\;2020\;(January\;-\;August)}\;x100$$

The expected value for the event was calculated considering the average of the past five years, prior to the occurrence of the COVID-19 pandemic (i.e. from 2015 to 2019), as recommended.^9^ The results were expressed as percentages, such that positive values indicated excess numbers of cases, and negative values indicated decreased numbers of cases.

This study did not require any adjudication from a research ethics committee, since it used data from the public domain.

## RESULTS

**Figure 1.**
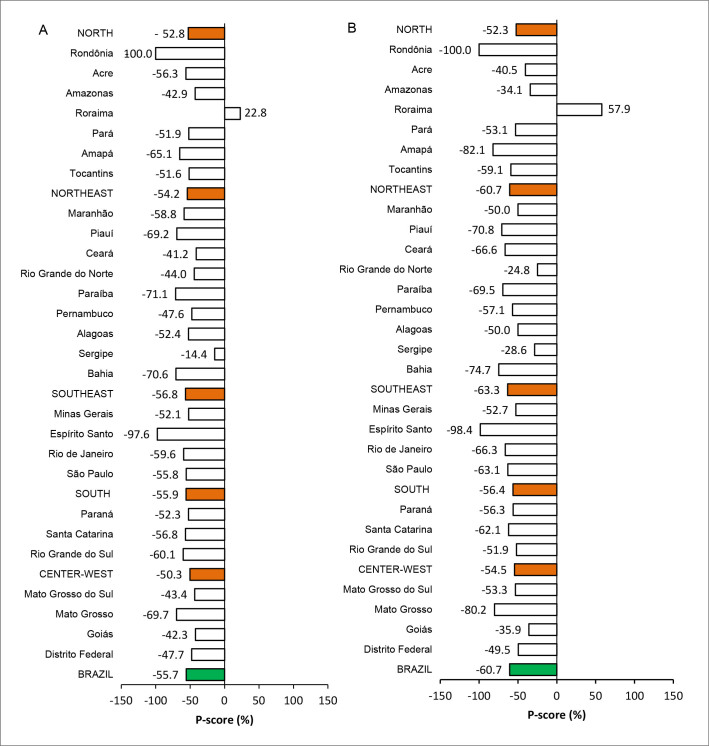
P-scores from reporting of meningitis (A) in the general population and (B) among children under 15 years of age. Brazil, regions and states. 2020.

**Figure 2.**
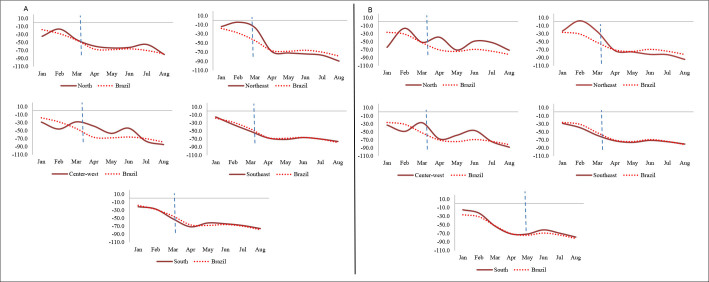
P-score curves from reporting of meningitis in the general population (A) and among children under 15 years of age (B). Brazil and regions; 2020.

From January to August 2020, 4,712 cases of meningitis were reported nationwide in the general population. For this same year, according to the calculation of mean values, 10,634 notifications were expected nationwide, thus resulting in a P-score of -45.7%. Considering only meningitis notifications among children under 15 years of age, 2,101 cases (44.6%) were recorded in 2020, while 5,343 notifications were expected nationally, thus giving rise to P-score of -60.7% for this group (Figures 1 and 2).

The southeastern region of Brazil had the highest number of notifications (2,446 registered cases; 51.9%). Out of this total, 1,131 notifications were from children under 15 years old (46.2%). In the southeastern region, in both groups, the P-score was negative, since 5,658 records were expected in the general group and 3,081 notifications in the under-15 group (P-scores of -56.8% and -63.3%, respectively). In addition, all other regions also showed negative P-scores in both groups, with declines of less than -50% (Figures 1 and 2).

The P-score curve showed a continuous drop in monthly notifications, in both groups, in all regions of the country after the beginning of the pandemic. In the first two months of 2020, before the arrival of COVID-19 in Brazil, there were also reductions in case notifications: -18% in January and -28.1% in February in the general group; -64% and -17% in the under-15 group. From March on, after confirmation of the first case of COVID-19 in Brazil, the drops were more pronounced, both nationally and regionally. The largest reductions were observed in August, just after the peak of the first pandemic wave, which was in June/July (-78% in the general group and -81.8% in the group with children under 15 years of age) (Figure 2).

Regarding the states, with the exception of Roraima, in the north of the country, which showed a positive P-score (22.8% in the general population and 57.9% in the group with children under 15 years of age), the other states showed declines in the numbers of notifications, especially Rondônia (no records in SINAN in 2020; P-score -100.0%) and Espírito Santo (three records in January 2020, among which one was in a child under 15 years of age; P-scores of -97.6% for the general population and -98.4% for children under 15 years of age). The state of Sergipe showed the smallest discrepancy between observed and expected values in the general population group (P-score of -14.4%) and Rio Grande do Norte was the state with values closest to what was expected for the group of children under 15 years old (P-score of -24.4%) (Figure 1).

## DISCUSSION

The negative effects of the pandemic on making diagnoses of other diseases have been reported worldwide.^3,4,6,10^ In Brazil, concerning only meningitis, the significant decrease in the number of compulsory notifications in 2020 does not seem to have occurred as a result of lower incidence of this disease during the pandemic period of COVID-19. As has been observed in relation to other diseases, this decrease in the number of records may be indicative of a reduction in the number of diagnoses made, or of operational losses that the pandemic has caused to the meningitis surveillance programs, in view of the guidelines provided by national and international healthcare organizations.^3,4^

Non-pharmacological measures to reduce transmission of COVID-19 may have influenced the chain of meningitis transmission in Brazil. The lower exposure engendered through isolation and social distancing actions during this period is one of the hypotheses for the drop in the rates, although we believe that this alone would not have the capacity to result in such a decline. Additionally, the pandemic caused changes to the routine of outpatient settings, including reductions in the numbers of consultations and absence of healthcare professionals infected by the virus, which would also have contributed to slowing down the number of diagnoses made.^4,11^ On the other hand, fear made the population afraid to attend hospitals and healthcare centers, with a consequent impact on notifications of many diseases, including meningitis.

It is noticeable that the instability caused by the pandemic hampered and still interferes with normalization of the healthcare network and data supplied to healthcare information systems. Timely feeding of data from the National Health System (Sistema Único de Saúde, SUS) into its databases is very important for enabling analysis and monitoring of the health conditions of the Brazilian population. If this does not take place, it could lead to increases in hidden prevalence and maintenance of the transmission chains of many diseases.^3^ Furthermore, the records in these systems make it possible to evaluate the impacts resulting from ongoing government projects, such as interventions and vaccination campaigns, as well as guiding the development of new healthcare policies.^12^

This study had limitations, among which its use of secondary data stands out. The quality of such data is influenced by the capacity of the local surveillance system. In addition, we can highlight the impact of the pandemic on the process of notification, typing, investigation and closure of cases.

## CONCLUSIONS

The pandemic caused by COVID-19 had a negative impact on reports of meningitis in Brazil in 2020, with a sharp and constant drop throughout all the months studied. Additionally, an inverse correlation was observed between cases of COVID-19 and reported cases of meningitis, especially in August, after the first peak of the pandemic in Brazil. This impact should be seen as a warning sign for political and healthcare authorities, and it highlights the need for improvements in healthcare and surveillance services, such as adoption and/or strengthening of strategies for ensuring diagnoses and mandatory notifications while the pandemic persists.

Furthermore, it is essential and urgent to investigate how COVID-19 can influence the prognosis of patients with meningitis, considering that there are no studies that address this aspect, and that this information can help prevent occurrences of the disease.
